# Impact of the first phase of COVID-19 pandemic on childhood routine immunisation services in Nepal: a qualitative study on the perspectives of service providers and users

**DOI:** 10.1186/s40545-021-00366-z

**Published:** 2021-09-29

**Authors:** Asmita Priyadarshini Khatiwada, Smriti Maskey, Nistha Shrestha, Sunil Shrestha, Saval Khanal, Bhuvan KC, Vibhu Paudyal

**Affiliations:** 1 Department of Pharmaceutical and Health Service Research, Nepal Health Research and Innovation Foundation, Lalitpur, Nepal; 2 University of North Texas Health Science Center, Fort Worth, TX USA; 3 Department of Pharmaceutical Outcomes and Policy, University of Florida, Gainesville, FL USA; 4 Division of Health Sciences, Warwick Medical School, University of Warwick, Coventry, UK; 5 School of Pharmacy, Monash University Malaysia, Jalan Lagoon Selatan, 47500 Bandar Sunway, Selangor Malaysia; 6 School of Pharmacy, Institute of Clinical Sciences, College of Medical and Dental Sciences, University of Birmingham, Birmingham, UK

**Keywords:** Immunisation, COVID-19, Nepal, Pandemic, Vaccination

## Abstract

**Background:**

The COVID-19 pandemic has disproportionately affected all essential healthcare services delivery in low-resource settings. This study aimed to explore the challenges and experiences of providers and users of childhood immunisation services in Nepal during the COVID-19 pandemic.

**Methods:**

Semi-structured qualitative interviews were conducted with childhood immunisation service providers and users (i.e., parents of children) from Kathmandu valley, Nepal. All interviews were conducted through phone or internet-based tools, such as Zoom, WhatsApp, and messenger. All interviews were audio-recorded, transcribed verbatim, and analysed using theme-based content analysis in an Excel spreadsheet.

**Results:**

A total of 15 participants (*n* = 7 service providers and *n* = 8 service users) participated. Six themes were identified, namely: (1) impact of COVID-19 and lockdown on childhood immunisation services; (2) motivation and resilience for childhood immunisation; (3) Biosafety practices and Personal Protective Equipment (PPE) availability during the COVID-19 pandemic; (4) service adjustments and guidelines during pandemic; (5) availability of vaccines; and (6) immunisation program resilience in view of COVID-19. Service providers mentioned facing disruptions in services and some parents had decided to delay scheduled immunisation. However, most service providers showed determinations to deliver the services with high morale, while most service users reported taking their children for immunisation. Families migrating from urban to rural areas during the pandemic led to service providers having no means to confirm complete immunisation of migrating children. Service providers also experienced lack of adequate guidance to deal with the pandemic and personal protective equipment to protect themselves and service users.

**Conclusion:**

Despite experiencing disruptions in childhood immunisation service due to the COVID-19 pandemic, service users and providers were determined to vaccinate the children. There is an urgent need for effective preparedness plans to be in place to address the observed barriers and to ensure resilient immunisation services during ongoing and future pandemics.

**Supplementary Information:**

The online version contains supplementary material available at 10.1186/s40545-021-00366-z.

## Background

The Coronavirus infectious disease (COVID-19) pandemic has exhibited collateral challenges to the healthcare systems globally [1]. By the mid of July 2021, the global cases of COVID-19 had exceeded 171 million with more than 3 million deaths [2]. Total cases in Nepal during that time was 658,778 cases with 9412 deaths [2]. To this date, there lacks an effective antiviral agent against the causative agent, SARS-CoV-2. Therefore, public health measures, such as social distancing, lockdowns, hand hygiene, contact tracing, quarantine, and isolation remain the mainstay of prevention and mitigation measures [3–5].

The Government of Nepal imposed a nationwide lockdown on 24th March 2020 after the second case of COVID-19 was confirmed in Nepal on 23rd March 2020 [6, 7]. This lockdown came as an early response to the pandemic in Nepal and went through different phases, including the closure of internal and external transportation, businesses, and academic institutions. In addition, the hospitals and healthcare facilities had limited capacity to provide essential health services with inadequate provision of personal protective equipments (PPEs) for healthcare providers and the fear of contracting the virus [5, 8]. As a result, the lockdown was lifted in September 2020, with a subsequent wave of COVID-19 commencing April 2021 [9].

In addition, the lockdown measures have been suggested to impact Nepal’s decade-long substantial progress on child health, especially the routine childhood immunisation services. Nepal's routine childhood immunisation services were suspended in different parts of the country, resulting in approximately three million children aged 9 months to 5 years missing their regular vaccination doses [10, 11].

In Nepal, routine childhood immunisation is conducted under the National Immunisation Program implemented by the Child Health Division of the Department of Health Services. Under this program, a total of 11 antigens are provided to children throughout the country, including those of geographically, economically hard to reach, marginalised communities through district hospitals, primary healthcare centers and health posts [12]. The immunisation services are delivered through all government health facilities, including outreach programs and mobile clinics in communities (VDCs and municipalities) all over the country in a periodic manner. In addition to these, immunisation services are provided at private hospitals, Non-governmental Organisations (NGOs)/International non-governmental organisations (INGOs), medical colleges/teaching hospitals, and district ward offices [13]. Among various members of the healthcare delivery system, Female Community Health Volunteers (FCHVs), vaccinators, immunisation officers, Auxiliary Nurse-Midwifes (ANM), and immunisation supervisors are key members involved in the routine childhood immunisation in health posts, outreach clinics and during the community vaccination campaigns [14].

Measles–Rubella (MR) vaccination is among many other vaccines included in the national immunisation schedule for children in Nepal [13]. The pandemic hit Nepal when the Government of Nepal set to conduct the MR vaccination campaign in two phases to eradicate measles and rubella from mid-February to mid-April 2020, including oral polio vaccine in selected regions. As a result, the campaign was completed in some places before the COVID-19 cases were seen in Nepal [15]. However, the rise in the cases of COVID-19 resulted in the cancellation of the MR vaccination campaign in many districts of the country [10].

Consequently, an outbreak of measles was reported in Dhading and Gorkha districts with 160 cases [16]. Therefore, this study was conducted in Kathmandu, Bhaktapur and Lalitpur districts, where the MR campaign was postponed due to lockdowns and rise in COVID-19 cases, and the immunisation service was restarted amid lockdown [17, 18]. Therefore, to better capture the experiences of the service providers and users of the immunisation services, these three districts of the Kathmandu valley were considered for the study.

Like other South-East Asian countries, the Government of Nepal adopted mitigation efforts such as preventive and hospital-based interventions based on the guidelines provided by World Health Organization (WHO) to tackle the COVID-19 pandemic. However, little is known about how the COVID-19 pandemic affected the immunisation service delivery and utilisation among the Nepalese population [19]. To the best of our knowledge, this is the first qualitative study that aims to explore the perceptions and the experiences of service providers and users of childhood immunisation services during the COVID-19 pandemic in Nepal.

## Methods

This qualitative study was conducted to explore childhood immunisation services during the COVID-19 pandemic in Kathmandu valley in Nepal.

### Study setting

This study was conducted in Kathmandu valley, including three districts, namely, Kathmandu, Bhaktapur and Lalitpur of Bagmati Province in Nepal. Kathmandu city is the capital city of Nepal. The population of Kathmandu valley is around 2 million. Kathmandu city stands at an elevation of 1400 m above sea level and lies in the warm temperate zone. Kathmandu valley is the main administrative and business center of Nepal. It is the most developed, well-networked, and densely populated place in Nepal. Most of the tertiary care hospitals, specialist health centers, and private hospitals are in Kathmandu Valley. Because of the economic opportunities, people from all over Nepal come to Kathmandu Valley for employment, business, studies, and other prospects. The demography of Kathmandu Valley comprises people from various socioeconomic and ethnic groups.

### Research participants

In the case of service providers, the initial list of potential participants was prepared via the review of the official database of the Ministry of Health and Population (MoHP), National Immunisation Programme and the District Health Offices (DHOs) of Bagmati province. The participants were then called via telephone and informed about the study, and those who were willing to participate in this study were invited and scheduled for an interview. The potential service users were identified and contacted based on network of the researchers. Due to limitations brought upon by the pandemic, all the respondents in this study were from Bagmati province. All potential participants were contacted by email, social media, and phone. Once all the potential participants were contacted, the research participants were recruited using snowball sampling method. The service users in this study are the parents/caregivers who got their children (0–2 year age) vaccinated during COVID-19 pandemic, and the service providers refer to those delivering the immunisation services, including FCHVs, ANMs, immunisation supervisor and officer, and cold chain consultant. Participants needed to be 18 years or older, be a permanent resident and/or working in the study geography, and be able to respond over a phone call, Zoom, WhatsApp, or messenger call for the interview (Table 1).

**Table 1:** Demographic details of research participants

Participant ID	Gender	Highest level of education completed	Occupation	Years of experience in the current designation	Mode of Interview	Duration of interview (in min)
IDI_SP-01	Female	Grade 9	FCHV	26	Telephone	60
IDI_SP-02	Female	Grade 10	FCHV	28	Telephone	75
IDI_SP-03	Female	Grade 10	FCHV	20	Telephone	65
IDI_SP-04	Female	Grade 10	Senior ANM	3	Messenger	60
IDI_SP-05	Male	Graduate level	Cold chain consultant	2	WhatsApp	85
IDI_SP-06	Male	Graduate level	Immunisation officer	11	Telephone	79
IDI_SP-07	Male	Graduate level	Immunisation supervisor	24	Messenger	24
	*Details of service users (parents)*			*Number of children*		
IDI_SU-01	Female	High school	Housewife	1	Telephone	30
IDI_SU-02	Female	Graduate level	Housewife	1	Zoom	29
IDI_SU-03	Female	Graduate level	Housewife	1	Zoom	30
IDI_SU-04	Female	Graduate level	Housewife	1	Zoom	20
IDI_SU-05	Female	Graduate level	Lecturer	1	Messenger	25
IDI_SU-06	Female	Graduate level	Unemployed	1	Zoom	21
IDI_SU-07	Male	High School	Business	1	Messenger	25
IDI_SU-08	Male	Graduate level	Multimedia producer	1	Messenger	20

### Data collection

Semi-structured qualitative interviews were conducted to collect data from August 2020 to December 2020. The semi-structured interviews allowed the interviews to be iterative and allowed interviewers to ask open-ended questions based on interview guidelines while remaining focused on the main research topic [20]. Given the risks and restrictions posed by the COVID-19 pandemic, the data were not collected in person for the safety of the interviewer and the participants. Therefore, the interviews were conducted virtually via phone or video conferencing as per the convenience of the participants [21]. Potential participants were contacted and informed about the study. After they consented to the interview, an appointment was arranged for the interview. The second call was made on the agreed date and time to conduct the interview. All interviews were audio-recorded after receiving verbal consent to do so. The interview recordings were transcribed verbatim. The interview transcript was read and re-read by the interviewer before the following interview. The interviews were refined in an iteration as per the information from previous interview transcript. The saturation was observed around 14 interviews when no new information was noted. A total of 15 research participants was interviewed for this study. Interviews lasted for an average of 64 min with service providers and 25 min with service users.

APK, SS and BKC conducted the interviews based on the pretested interview guidelines developed by SK, NS, VP and SM. The interview guide was developed considering the building blocks of the health system, including leadership and governance, service delivery, financing, health workforce, medical products, vaccines, technologies, and health information systems [22]. The interview guide was reviewed by experts comprising two public health officers and one public health physician. Furthermore, a pilot interview was carried out with two participants, based on which the interview guide was finalised. The interview guidelines intended to explore the participant’s general experience of COVID-19 pandemic, health care seeking behaviour, human resources availability, immunisation services availability, accessibility, and utilisation (Additional file 1: interview guidelines). All interviews were collected in the Nepali language, and the interviewers were guided by SS, NS, SM, SK, BKC, VP, who have experience in collecting and reporting qualitative data.

All interviews were recorded on mobile phones or laptops (in video conferencing) and saved in a password-protected folder. Interviews were transcribed in Nepali and translated into English by all authors fluent in English and Nepali. All the authors cross-checked each other’s translations for completeness and accuracy. The translated versions of the transcripts were used in the analysis.

### Data analysis

Data were analysed using theme-based content analysis in MS Excel spreadsheet. Recorded interviews were transcribed by researchers APK, SM, NS and SS. The transcript was re-read and reviewed by SS, NS, SM and SS to verify the accuracy of the transcripts. The transcript was ready for analysis once approved and verified by the researchers. The researcher BKC undertook a thematic analysis of the raw data to look for the relevant themes/issues. These themes/issues were discussed by BKC, SM, SS, APK, SK, VP and NS initially and then with other researchers (authors) who were not involved in transcribing and analysing the transcripts to ensure that it is reliable and trustworthy. All researchers read each transcript multiple times to ensure that themes/issues correctly present the transcript's content. Emergent issues/themes were discussed with all researchers. All researchers agreed to the final themes/issues of the content analysis. To minimise the influence of researchers’ values and opinions on data analysis and theme building, the researchers took special care to be critically self-reflective about their preconceptions and analytical focus. Iterative discussion within the research team also minimised any bias.

### Ethical considerations

The ethical approval for this study was obtained from the institutional review committee (IRC) of Nobel College, Kathmandu, Province Bagmati, Nepal. We informed all participants that their participation in this study was voluntary and could withdraw anytime. All participants were well informed about the study objectives, benefits and harms and were encouraged to ask questions at any interview stage. The verbal consents from each participant were audio recorded. At all stages of the study, the confidentiality and anonymity of the research participants were maintained.

## Results

The content of the result were analysed using simple thematic analysis. The findings from this qualitative research were categorised into six general themes: (1) impact of COVID-19 and lockdown on childhood immunisation services; (2) motivation and resilience for childhood immunisation; (3) biosafety practices and PPE availability during the COVID-19 pandemic; (4) service Adjustments and Guidelines during a pandemic; (5) availability of Childhood Vaccines; and (6) vaccination program resilience in view of COVID-19.

### Impact of COVID-19 and lockdown on childhood immunisation services

Most participants agreed that the pandemic had impacted the childhood immunisation service delivery and utilisation. In addition, most of the service providers mentioned that COVID-19 affected the delivery of some vaccines during the initial phase of COVID-19, along with the decrease in parents bringing their children for vaccinations.

Although the vaccine supply chain was not completely disrupted, the service providers echoed that the immunisation service was delayed and children missing vaccine doses were rescheduled and vaccinated later.

*“What I can tell from my experience of observing immunisation during COVID-19 is that during the initial phase very few of the parents came to the health facility for vaccination so there was a decline in uptake of vaccines…”* [IDI SP-05]

### Motivation and resilience for childhood immunisations

All study participants expressed fear and anxiety due to the COVID-19 pandemic as the number of cases increased, and COVID-19 progressed. Service providers highlighted that COVID-19 had affected all aspects of public life, including family and social life. Services users also voiced their fears and anxiety brought about by the COVID-19.

Despite the fear in society, service providers were determined to deliver the services with high morale, while most service users reported taking their children for vaccination. The service providers were motivated by their family members and moral obligation to continue providing services during the pandemic and lockdowns. In contrast, prior health education provided by the service providers regarding the importance of childhood vaccination motivated the service users to bring in their children for vaccination amid the global pandemic and lockdowns.

*“......Yet the second month of lockdown was very stressful to me. I was worried how long everything would remain close. I heard many rumors like it will last till Dashain (October-November 2020), some said the schools will remain close till Shrawan (July-August 2021). But I still had hope that things would work out, so I was very happy when my husband was supportive of my work.”* [IDI_SP-01]

*“When I was first asked to come to work during the lockdowns, I was really scared because ma’am said I had to come in all circumstances. So, I discussed with my husband. Even my family members said that working in the health sector, I had to work more during such public health situations. They assured me that if I were careful and used sanitisers, gloves, and masks properly, I would not be infected….”* [IDI_SP-02]

At the societal level, all stakeholders reported migration of the families who came to the city for work back to their villages when there was partial ease on the lockdowns. The service providers were convinced that those children would be vaccinated at the local health facilities in their respective villages. However, there was no means to confirm that the children who migrated back to their village with their families were vaccinated as scheduled.

### Biosafety practices and PPE availability during the COVID-19 pandemic

The overall biosafety practice was poor and inadequate, as observed by participants. Participants reported lack of adequate supply of PPE kits for healthcare workers. Essential PPE supplies, such as gloves, PPE kits, and visors were not in sufficient quantity.

The health system did not properly cushion service providers who were at the forefront of vaccination delivery. As per the service provider, they lacked adequate PPE, facilities, and other support, putting them at risk of getting infected by COVID-19. Furthermore, their concerns were not appropriately addressed by the higher management.

*“So, when we were going house to house to find the people who recently came back from abroad for the PCR tests, we were given a pair of gloves and masks, which did not even last for the entire day. So, the next day we used our own personal gloves and masks. During the Rubella and Measles vaccination program, we were given only masks but not gloves or sanitisers, so we used our own gloves and sanitisers. There were sanitisers for the vaccinators but not us, so we used our own. During the Vitamin A program as well, they said that we did not need any gloves or sanitisers. So, we bought it ourselves.”* [IDI_SP-02]

Health service users noted a lacklustre in terms of Standard Operating Procedure (SOP) compliance. The government of Nepal has put forward to continue the childhood immunisation services amid COVID-19 pandemic by taking necessary precautionary measures, such as physical distancing and using protective and safety gears [23]. However, they observed lapses in fever screening and social distancing measures.

Despite the inadequate PPE supplies, service providers felt they did their best to deliver vaccination while following COVID-19 SOPs safely.

“…again from the institutional side what I noticed that everyone was afraid of COVID-19, there were not adequate PPE.” [IDI_SP-05]

### Service adjustments and guidelines during pandemic

In general, the anticipation and planning of immunisation service delivery during COVID-19 pandemic were inadequate. Service providers reported that there were not any clear guidelines on how to carry out immunisation efforts during COVID-19 pandemic. Furthermore, all the stakeholders at the peripheral level health facilities felt lapses in communication, mobilisation of healthcare staff, and adequate PPE for front line staff.

FCHVs who manage the delivery of vaccination felt that they lacked reassurance and safety cushioning during COVID-19 pandemic. On the health system side, participants felt that anticipation of healthcare staff’s extra workload and impact of COVID-19 pandemic on delivery of regular health service was not properly planned during COVID-19 pandemic because of which delivery of these regular health services too was affected during COVID-19 pandemic.

*“So, when we were going house to house to find the people who recently came back from abroad for the PCR tests, we were given a pair of gloves and masks, which did not even last for the entire day. So, the next day we used our own personal gloves and masks. During the Rubella and Measles vaccination program, we were given only masks but not gloves or sanitisers, so we used our own gloves and sanitisers. There were sanitisers for the vaccinators but not us, so we used our own. During the Vitamin A program as well, they said that we did not need any gloves or sanitisers. So, we bought it ourselves.”* [IDI_SP-02]

### Availability of childhood vaccines

The service providers stated that vaccine logistics was not affected during the COVID-19 pandemic except during the early chaos of COVID-19 pandemic. The public health officials said that the vaccine flow from central store to regional store and then to district level store was not hampered as health logistics and essential commodities were allowed even during lockdown.

The public health officers said that the Department of Health Services managed the vaccination and health logistics and health staff, but the local government's delivery, timing, vaccination site, etc., is done by the local government in consultation with the local health facility.

*“No sir ....so far as I know… there was not any problem with the supply of vaccines… there were enough vaccines at the district store, and the health post or primary health center got it….and, yes…even during COVID-19, the movement of vaccines and health commodities were allowed.”* [IDI_SP-07]

### Vaccination program resilience in view of COVID-19

Service providers believed that an established program should have a proper structure and plan, experienced and trained workers, strong logistics, and a good network of dedicated frontline health workers. Furthermore, service providers stated that they spread awareness regarding vaccination, provide information regarding the date and site of vaccination.

However, service providers said that not having proper guidelines on delivering vaccines during COVID-19 pandemic, lack of adequate resources support from local government, and a lack of proper database tracking on health care services utilisation in the urban area are some of the weaknesses of the vaccination program amid COVID-19 pandemic.

*“What I have seen is that our vaccination program is good… it has an established program with years of experience, we have proper plans at all levels on what to, who will do what, we have a proper schedule…. And not to forget the strong supply chain and store of vaccine and health supplies that we maintain at our stores…. we have a good network of volunteers… (FCHVs)….these volunteers are our backbone… They help in the delivery of vaccines….they pass the message about the vaccine campaign to local places and parents…they are in touch with parents in their local area….they are a strong resource….that’s how we were able to vaccinate children even during COVID-19…. I myself have observed vaccinations in different regions this time… it’s due to this backup that we were able to do vaccination even during this tough time…”* [IDI_SP-05]

## Discussion

The COVID-19 pandemic has affected and disrupted the health services delivery at all levels of the Nepalese health system. Immunisation is an essential health service delivery for every country. The addition of new vaccines and immunisation schedules, a complex supply chain involving multiple stakeholders at an international and national level, cold chain infrastructure and public healthcare delivery structure and process makes immunisation a micro-system in itself [24]. Effective management of immunisation both during normal and disaster time shows the resilience and strength of the health system. In this present study, we explored delivery of the national immunisation service during COVID-19 pandemic and the nature and extent of impact of COVID-19 pandemic on childhood immunisation services.

Overall, our study shows that despite many barriers, both providers and users perceived that childhood immunisation service delivery continued during the COVID-19 pandemic; however, there was a disruption of services at specific points/periods during COVID-19 pandemic. There was an attempt to trace those children who missed their regular immunisation during the COVID-19-related disruption by the service providers. Despite such attempts, a lack of a real-time dynamic immunisation database and temporary migration and movement of families during COVID-19 pandemic affected the tracing of all the children missing immunisation.

The members involved in immunisation delivery at the peripheral level and the parents reported inadequate biosafety practice during COVID-19 pandemic. They highlighted the issues such as lack of PPE, poor compliance to COVID-19 SOP measures by the service users. In addition, the inadequate healthcare system that is malign with infrastructure shortage and lack of effective management and a lackadaisical attitude regarding the health service delivery during pandemic might have contributed to inadequate preparedness and cushioning of the health system and healthcare staff during the COVID-19 pandemic [25, 26].

In the current study, even though the childhood immunisation service deliveries were affected during the early phase of COVID-19 pandemic in Nepal, the ground level members of the immunisation team, i.e., FCHVs, ANMs were delivering the service in the later period. In addition, although the regular immunisation service and the mass immunisation campaign were continued intermittently, taking appropriate precautionary measures, only a smaller number were brought for vaccination shots because of the fear of COVID-19 transmission. Almost consistent findings were observed in Saudi Arabia and the United States, where the reasons for low vaccine coverage were the fear of contracting COVID-19 at healthcare facilities, restrictions in movements for the public, limitations within healthcare facilities, and unavailability of vaccination services during lockdown [27, 28]. Indeed, the additional potential reasons mentioned above may have led to fewer children attending the vaccination service in Nepal. In addition, in case of the current study, this might also be due to lack of effective communication about the operation of vaccination centers during COVID-19 pandemic. The effective tracking of the missed children and administering the missed doses of vaccines is crucial. Failure to do so might lead children to the resurgence of vaccine-preventable diseases (VPDs) [29]. A study in Africa has reported that the benefits of routine childhood vaccination outweigh the risk of COVID-19 transmissions and death and focused on continuing the vaccination process amid COVID-19 pandemic [30].

The service providers reported a lack of specific guidelines on child vaccination during COVID-19 and communication gaps and issues in mobilisation of the healthcare workforce. The guidelines for conducting routine childhood immunisation safely during the COVID-19 pandemic have been developed by organisations such as Centers for Disease Control and Prevention (CDC), and WHO [31, 32] and countries such as New Zealand [33] have implemented their version of guidelines to follow for vaccination during COVID-19. However, a tailored guideline focusing on conduction of the child vaccination in Nepal seems to lack action, which might have led to chaos and immunisation service disruption. Nevertheless, this study showed a need to adopt such guidelines for Nepal and effectively implement and communicate them to all health service delivery levels.

In this study, the FCHVs reported that reassurance from colleagues was not present during COVID-19 pandemic. In Nepal, the vaccination programs greatly rely on FCHVs, and they were vulnerable to higher exposure to COVID-19 due to added burden in service delivery. In addition, the top-down bureaucratic culture that does not consider the concerns of the peripheral level service delivery staff might have led to such a situation. The front-line peripheral level staff such as the FCHVs and the ANMs are essential part of immunisation services, and the health system needs to cushion these precious staff from such exposure. The health system needs to be reassuring and use all the available resources at its disposal to create a safe working environment for such staff during COVID-19 and any future pandemics.

It was reported that the family and social life of the healthcare workers were affected much because of COVID-19 in the current study. A scoping review showed that the healthcare workers faced emotional challenges and stress in dealing with the patients and carrying out their regular functions amid COVID-19 pandemic [34]. In addition, it was observed that transportation restrictions, fear of being exposed to virus, illness, paucity of PPEs and ineffective infection prevention and control were reasons for insufficient healthcare workers in different countries [35–37]. Furthermore, even though healthcare workers were trying their best to provide services from their end, literature shows the experience of social stigma against healthcare workers in different countries, including Nepal [38]. However, no participants reported this as an issue in our study.

Our findings showed that parents realised the importance of vaccination and brought their children for vaccination despite COVID-19 fear. These parents also tried to stick to the COVID-19 safety measures. This result was similar to a study conducted in Saudi Arabia, where parents' positive attitudes about child vaccination were observed [27]. In contrast, another study in Saudi Arabia reported that many parents were not concerned about vaccination delay [39]. The variation in results may be associated with the awareness level of parents, since a significant association between positive attitude towards vaccination and parents’ education was reported [40].

This study also observed that many children who migrated from their temporary stay to permanent addresses during COVID-19 pandemic got their scheduled vaccines in their respective villages. The participants did not report the negative impact on vaccine logistics. Even during the lockdown, public health officials hindered the vaccine delivery from central to district stores. In lack of PPE, delivery of some of the regular healthcare services were affected at local levels, but the local governments of that specific areas took care of the proper vaccine delivery and distribution at local levels. In contrast to this result, in astudy in Africa,  it was observed that the COVID-19 causes logistical constraints to vaccine supply and delivery to the service points [11]. The undisturbed logistics to vaccine supply and delivery in Kathmandu valley may be because of the availability of sufficient vaccines required within the country for the time being and no travel restrictions for vehicles carrying vaccines. It could also be because our study site was near the district store of vaccines. Hence, one of the limitations of this study is that we cannot generalise the findings of this study to the entire country.

From a health system perspective, childhood immunisation service delivery must be adapted to COVID-19 safety (biosafety) requirements and other future disasters and pandemics. Health services delivery during the pandemic and other disasters is challenging; however, essential health services such as immunisation need to continue even during a pandemic. The biosafety measures were inadequate, and there were lapses in the supply of PPE and other health supplies, proper training of the health workers, operation guidance and proper cushioning of healthcare workers, especially the front line and much vulnerable FCHVs. Furthermore, the lack of a real-time immunisation database and inability to track the number of families migrating temporarily during COVID-19 pandemic, especially from urban areas, also shows the need to improve database tracking and effective linking of local population database to the national childhood immunisation program. Despite the tremendous pressure from COVID-19 on health service delivery, the logistics aspect of the national immunisation program kept the vaccine supply chain ongoing. It shows the strength of the national immunisation program and continuous effort of all the stakeholders towards strengthening the program. Nepal should build on this strength of the national immunisation program and address the system (supply) side inadequacies for a sustainable childhood immunisation program.

## Conclusion

Despite experiencing disruptions in childhood immunisation service due to the COVID-19 pandemic, service users and providers were determined to vaccinate the children. There is an urgent need for effective preparedness plans to be in place to address the observed barriers and to ensure resilient immunisation services during ongoing and future pandemics. Service guidance needs to be developed to address any disruptions in future pandemics that can address service provider and user barriers, anxiety and fears and the mechanisms to trace children and families that migrate during the pandemic to ensure complete immunisations.

### Limitation

Convenience sampling was used in the study, so there is a probability that some potential information source might have been missed. In the current study, low-income service users were not covered, leading to incomplete information about the real-world hardships among different service users. In addition, there is a possibility of recall bias in this study, since participants were asked some retrospective questions. Furthermore, the study was carried out in Kathmandu Valley, Nepal's central administrative center. The infrastructure and logistics are better in Kathmandu Valley and its periphery compared to Nepal’s rural areas. Therefore, the findings of this study may not be generalisable to the rest of Nepal, and it is also one of the limitations of this study.

## Supplementary Information

**Additional file 1.** Interview guideline.

## Data Availability

All the data and materials are available on request to the corresponding author.
