# Supplementary material for: Impact of the first phase of COVID-19 pandemic on childhood routine immunisation services in Nepal: a qualitative study on the perspectives of service providers and users
Source: J Pharm Policy Pract. 2021 Sep 29;14:79. doi: 10.1186/s40545-021-00366-z (PMC8479266; doi:10.1186/s40545-021-00366-z)
Supplement: Supplementary file 1 — Additional file 1. Interview guideline. [file 40545_2021_366_MOESM1_ESM.docx]

**KII interview guidelines Policy level- Draft V2.7.22.2020**

| Name of the interviewee: |  |
| --- | --- |
| Designation/Title/Position: |  |
| Educational Qualification: |  |
| Years of work experience: |  |
| Years of work experience in the current designation: |  |
| Name of the interviewer: |  |
| Date: |  |
| Time: |  |
| Start time: |  |
| End time: |  |
| How was the interview conducted? | Phone call / Zoom / Viber / Messenger /  WhatsApp / In-person |

|  | Guides |
| --- | --- |
| Icebreaker | 1. What is your opinion on the current COVID-19 pandemic? 2. How has it affected you? How has it changed the way you do your job? |
| Service Delivery | 1. Can you describe the regular situation of immunization services in your area before the start of COVID-19?    1. What are the regular vaccines provided?    2. When are regular immunization services delivered?    3. To whom? How? Probe about outreach clinics/mass vaccine campaigns, number of days immunization services/outreach services are provided 2. Has COVID-19 affected the delivery of regular immunization services? How?    1. In your opinion, is it important to provide regular immunization services during a pandemic situation like this? Why? Why not?    2. How are the regular immunization services being delivered? Who takes the responsibility? What are the concerns from the parents and health care workers? How do you address it?    3. How was the recent/latest mass vaccination campaign implemented? What was the response from the parents?    4. What special precautions (taking temperature of everyone in the health facility/ social distancing/ use of PPEs) or provisions have been put in place to prevent staff and health service users from COVID-19.    5. What were the challenges faced? How did you address the challenges? |
| Health Workforce | 1. Can you explain generally who is involved in providing immunization services?    1. In your opinion, are the current number of people sufficient for providing immunization services? 2. How has COVID affected the health workforce for providing immunization?    1. Are there any Health workers/FCHVs who dropped out from immunization roles due to COVID? Why? Why not? How were such challenges (staff’s security issues and unwillingness to work) managed?    2. How are the staff managed during COVID-19? (rotation, change of duties, etc.) |
| Health information System/Resources | 1. What resources are you using/following for educating patients or running awareness regarding COVID-19 nationwide?  2. What resources or guides do you use as a safety measure during COVID-19? (guide regarding social distancing, hand washing, masks etc.)  3. Can you describe the Health Information Management System to manage vaccine logistics during normal time and during COVID-19? |
| Access to Vaccines | 1. Can you please elaborate about how the vaccines are made available in this facility/______ (place where they work)?  a. When? How often? Cold chain maintenance?  2. Was the vaccine supply chain affected due to COVID?  a. Any transportation issues due to travel restrictions?  b. Shortage of vaccines in the higher supply organization? |
| Financing | 1. Has COVID affected the funds allocated for providing regular immunization services? Why? Why not?  a. Were any additional funds allocated for providing services/managing health care workers/precautionary measures during COVID-19?  b. Were any additional funds allocated for maintaining supply chain during COVID-19? |
| Leadership/governance | 1. Has the Ministry of Health taken any steps to safely regulate immunization services during a pandemic?   a. any guidelines developed for local level health workers/FCHVs on how to safely regulate immunization services during pandemic?  b. What was the process of developing those guidelines? Who was involved?  c. Was there any involvement of representatives from national and international non-governmental bodies like WHO, SAVE the Children or UNICEF or any UN agencies? How were they involved; providing technical assistance to develop guidelines, proving human or financial resources?  d. Could you briefly explain about the guidelines? What new ways are explained? |
| Recommendations/Way forward | 1. What should be the way forward in terms of preparing health care workers/FCHVs and the health systems to provide regular immunization services during a pandemic or any epidemic in future? 2. In your opinion, what could have helped you address the challenges faced due to COVID, especially in delivering regular immunization services? 3. Are there any other things about immunization services and pandemics that you would like to add? |

Thank you for your time and participation

**KII guidelines for DPHO/DHO/PHCC medical officer**

| Name of the interviewee: |  |
| --- | --- |
| Designation/Title/Position: |  |
| Educational Qualification: |  |
| Years of work experience: |  |
| Years of work experience in the current designation: |  |
| Name of the interviewer: |  |
| Date: |  |
| Time: |  |
| Start time: |  |
| End time: |  |
| How was the interview conducted? | Phone call / Zoom / Viber / Messenger /  WhatsApp / In-person |

|  | Guides |
| --- | --- |
| Icebreaker | 1. What is your opinion on the current COVID-19 pandemic? 2. How has it affected you? How has it changed the way you do your job? 3. In the past few months, did you approach health service users who might be a suspected case of COVID-19? |
| Service Delivery | 1. Can you describe the regular situation of immunization services in your _______(district/municipality/village) before the start of COVID-19? 2. What are the regular vaccines provided? 3. When are regular immunization services delivered? 4. To whom? How? Probe about outreach clinics/mass vaccine campaigns, number of days immunization services/outreach services are provided   2. Has COVID-19 affected the delivery of regular immunization services? How?   1. In your opinion, is it important to provide regular immunization services during a pandemic situation like this? Why? Why not? 2. How are the regular immunization services being delivered? Who takes the responsibility? What are the concerns from the parents and health care workers? How do you address it? 3. How was the recent/latest mass vaccination campaign implemented? What was the response from the parents? *(probe as much details as you can in this question- how was it planned, challenges, facilitators, precautions, results, use of PPEs, social distancing, choice of place, use of social media for advertisement)* 4. What special precautions (taking temperature of everyone in the health facility/ social distancing/ use of PPEs) or provisions have been put in place to prevent staff and health service users from COVID-19 especially when providing immunization services? 5. What were the challenges faced? How did you address the challenges? |
| Health Workforce | 1. Can you brief us about who is involved in providing immunization services?    1. How many people? What capacity/role?    2. In your opinion, were the current number of people sufficient for providing the services? 2. Can you brief us about your role in providing immunization services? 3. How has COVID affected the health workforce for providing immunization?    1. Are there any Health workers/FCHVs who dropped out from immunization roles due to COVID? Why? Why not? How were such challenges (staff’s security issues and unwillingness to work) managed?    2. How are the staff managed during COVID-19? (rotation, change of duties, etc.) |
| Health information System/Resources[[15]](https://liveunthsc-my.sharepoint.com/personal/sm1358_live_unthsc_edu/Documents/Smriti-Personal/Immunization%20Paper/IDI%20guidelines_7.22.2020.docx#_msocom_15) | 4. What resources are you using/following for educating patients or running awareness regarding COVID-19 nationwide?  5. What resources or guides do you use as a safety measure during COVID-19? (guide regarding social distancing, hand washing, masks etc.)  6. Can you describe the Health Information Management System to manage vaccine logistics during normal time and during COVID-19? |
| Access to Vaccines | 1. Can you please elaborate about how the vaccines are made available in this facility/______ (place where they work)?[[16]](https://liveunthsc-my.sharepoint.com/personal/sm1358_live_unthsc_edu/Documents/Smriti-Personal/Immunization%20Paper/IDI%20guidelines_7.22.2020.docx#_msocom_16)  a. When? How often? Cold chain maintenance?  2. Was the vaccine supply chain affected due to COVID?  a. Any transportation issues due to travel restrictions?  b. Shortage of vaccines in the higher supply organization?[[17]](https://liveunthsc-my.sharepoint.com/personal/sm1358_live_unthsc_edu/Documents/Smriti-Personal/Immunization%20Paper/IDI%20guidelines_7.22.2020.docx#_msocom_17) |
| Financing | 1. Has COVID affected the funds allocated for providing regular immunization services? Why? Why not?[[18]](https://liveunthsc-my.sharepoint.com/personal/sm1358_live_unthsc_edu/Documents/Smriti-Personal/Immunization%20Paper/IDI%20guidelines_7.22.2020.docx#_msocom_18)  a. Were any additional funds allocated for providing services/managing health care workers/precautionary measures during COVID-19?  b. Were any additional funds allocated for maintaining supply chain during COVID-19? |
| Leadership/governance | 1. Has the Ministry of Health taken any steps to safely regulate immunization services during a pandemic?   a. Has the Ministry of Health developed any guidelines for local level health workers/FCHVs on how to safely regulate immunization services during pandemic?  b. *If yes, ask about the steps and guidelines;* Could you briefly explain about the guidelines? For whom? What new ways are explained?  *If no*, have you taken any steps to safely regulate immunization services during this pandemic in your district/region?  a. What was the process of developing those guidelines?   - 1. Who was involved?   2. Was there any involvement of representatives from national and international non-governmental bodies? How were they involved; providing technical assistance to develop guidelines, proving human or financial resources?   3. Could you briefly explain about the guidelines? For whom? What new ways are explained? |
| Recommendations/Way forward | 1. What should be the way forward in terms of preparing health care workers/FCHVs and the health systems to provide regular immunization services during a pandemic or any epidemic in future? 2. In your opinion, what could have helped you address the challenges faced due to COVID, especially in delivering regular immunization services? 3. Are there any other things about immunization services and pandemics that you would like to add? |

Thank you for your time and participation

IDI guidelines for health post in charge, AHW/ANMs

| Name of the interviewee: |  |
| --- | --- |
| Designation/Title/Position: |  |
| Educational Qualification: |  |
| Years of work experience: |  |
| Years of work experience in the current designation: |  |
| Name of the interviewer: |  |
| Date: |  |
| Time: |  |
| Start time: |  |
| End time: |  |
| How was the interview conducted? | Phone call / Zoom / Viber / Messenger /  WhatsApp / In-person |

|  | Guides |
| --- | --- |
| Icebreaker | 1. What is your opinion on the current COVID-19 pandemic? 2. How has it affected you? How has it changed the way you do your job? 3. As a frontline health care worker, how did you approach health service users who might be a suspected case of COVID-19? |
| Service Delivery[[19]](https://liveunthsc-my.sharepoint.com/personal/sm1358_live_unthsc_edu/Documents/Smriti-Personal/Immunization%20Paper/IDI%20guidelines_7.22.2020.docx#_msocom_19) | 1. Can you describe the regular situation of immunization services in your village/municipality before the start of COVID-19?    1. What are the regular vaccines provided?    2. When is the regular immunization services delivered?    3. To whom? How? Probe about outreach clinics/mass vaccine campaigns, number of days immunization services/outreach services are provided 2. Has COVID-19 affected the delivery of regular immunization services? How?    1. In your opinion, is it important to provide regular immunization services during a pandemic situation like this? Why? Why not?    2. How are the regular immunization services being delivered? Who takes the responsibility? What are the concerns from the parents and health care workers? How do you address it?    3. How was the recent/latest mass vaccination campaign implemented? What was the response from the parents? *(probe as much details as you can in this question- how was it planned, challenges, facilitators, precautions, results, use of PPEs, social distancing, choice of place, use of social media for advertisement)*    4. What special precautions (taking temperature of everyone in the health facility/ social distancing/ use of PPEs) or provisions have been put in place to prevent staff and health service users from COVID-19.  [[20]](https://liveunthsc-my.sharepoint.com/personal/sm1358_live_unthsc_edu/Documents/Smriti-Personal/Immunization%20Paper/IDI%20guidelines_7.22.2020.docx#_msocom_20) [[SM21]](https://liveunthsc-my.sharepoint.com/personal/sm1358_live_unthsc_edu/Documents/Smriti-Personal/Immunization%20Paper/IDI%20guidelines_7.22.2020.docx#_msocom_21)    5. What were the challenges faced? How did you address the challenges? |
| Health Workforce | 1. Can you explain generally who are involved in providing immunization services?    1. In your opinion, are the current number of people sufficient for providing the immunization services? 2. How has COVID affected the health workforce for providing immunization?    1. Are there any Health workers/FCHVs who dropped out from immunization roles due to COVID? Why? Why not? How were such challenges (staff’s security issues and unwillingness to work) managed?    2. How are the staff managed during COVID-19? (rotation, change of duties, etc.) |
| Health information System/Resources[[22]](https://liveunthsc-my.sharepoint.com/personal/sm1358_live_unthsc_edu/Documents/Smriti-Personal/Immunization%20Paper/IDI%20guidelines_7.22.2020.docx#_msocom_22) | 1. What resources are you using/following for educating patients or running awareness regarding COVID-19 nationwide?  2. What resources or guides do you use as a safety measure during COVID-19? (guide regarding social distancing, hand washing, masks etc.)  3. Can you describe the Health Information Management System to manage vaccine logistics during normal time and during COVID-19? |
| Access to Vaccines | 1. Can you please elaborate about how the vaccines are made available in this facility/______ (place where they work)?[[23]](https://liveunthsc-my.sharepoint.com/personal/sm1358_live_unthsc_edu/Documents/Smriti-Personal/Immunization%20Paper/IDI%20guidelines_7.22.2020.docx#_msocom_23)  a. When? How often? Cold chain maintenance?  2. Was the vaccine supply chain affected due to COVID?  a. Any transportation issues due to travel restrictions?  b. Shortage of vaccines in the higher supply organization?[[24]](https://liveunthsc-my.sharepoint.com/personal/sm1358_live_unthsc_edu/Documents/Smriti-Personal/Immunization%20Paper/IDI%20guidelines_7.22.2020.docx#_msocom_24) |
| Financing | 1. Has COVID affected the funds allocated for providing regular immunization services? Why? Why not?[[25]](https://liveunthsc-my.sharepoint.com/personal/sm1358_live_unthsc_edu/Documents/Smriti-Personal/Immunization%20Paper/IDI%20guidelines_7.22.2020.docx#_msocom_25)  a. Were any additional funds allocated for providing services/managing health care workers/precautionary measures during COVID-19?  b. Were any additional funds allocated for maintaining supply chain during COVID-19? |
| Leadership/governance | 1. Have you or other health workers (including FCHVS) been given guidelines on how to safely regulate immunization services during this pandemic?    1. How was it used?    2. Is it helpful? Why? Why not? What could have made it better? 2. Are you or other health workers (including FCHVs) provided with the personal protective equipment to safely provide the immunization services? 3. Are there any new governmental policies regarding service delivery during COVID-19? 4. Can you briefly tell me about how you feel regarding the preparedness measures taken by the higher authorities during this COVID?   a. Are there any mechanisms or system related to grievances to hear staff’s concern regarding COVID-19? How do you feel about that?  b. Are there any policies in place regarding health service delivery during emergencies? Has there been any new changes in policies regarding health service delivery during COVID-19?  c. Could you briefly explain about the guidelines? For whom? What new ways are explained? |
| Recommendations/Way forward | 1. What should be the way forward in terms of preparing health care workers/FCHVs and the health systems to provide regular immunization services during a pandemic or any epidemic in future? 2. In your opinion, what could have helped you address the challenges faced due to COVID, especially in delivering regular immunization services? 3. Are there any other things about immunization services and pandemics that you would like to add? |

Thank you for your time and participation

**IDI for FCHVs**

| Name of the interviewee: |  |
| --- | --- |
| Designation/Title/Position: |  |
| Catchup Area/Ward No of Service |  |
| Educational Qualification: |  |
| Years of work experience: |  |
| Name of the interviewer: |  |
| Date: |  |
| Time: |  |
| Start time: |  |
| End time: |  |
| How was the interview conducted? | Phone call / Zoom / Viber / Messenger /  WhatsApp / In-person |

|  | Guides |
| --- | --- |
| Icebreaker | 1. What is your opinion on current COVID-19 pandemic? 2. How has it affected you? How has it changed the way you do your job? 3. As a frontline health care worker, how did you approach health service users who might be a suspected case of COVID-19? |
| Service Delivery[[26]](https://liveunthsc-my.sharepoint.com/personal/sm1358_live_unthsc_edu/Documents/Smriti-Personal/Immunization%20Paper/IDI%20guidelines_7.22.2020.docx#_msocom_26) | 1. In your opinion, has COVID-19 affected the delivery of regular immunization services? How?    1. In your opinion, is it important to provide regular immunization services during a pandemic situation like this? Why? Why not?    2. How was the recent/latest mass vaccination campaign implemented? What was the response from the parents? *(probe as much details as you can in this question- how was it planned, challenges, facilitators, precautions, results, use of PPEs, social distancing, choice of place, use of social media for advertisement)*    3. What special precautions (taking temperature of everyone in the health facility/ social distancing/ use of PPEs) or provisions have been put in place to prevent staff and health service users from COVID-19.  [[27]](https://liveunthsc-my.sharepoint.com/personal/sm1358_live_unthsc_edu/Documents/Smriti-Personal/Immunization%20Paper/IDI%20guidelines_7.22.2020.docx#_msocom_27) [[SM28]](https://liveunthsc-my.sharepoint.com/personal/sm1358_live_unthsc_edu/Documents/Smriti-Personal/Immunization%20Paper/IDI%20guidelines_7.22.2020.docx#_msocom_28)    4. What were the challenges faced? How did you address the challenges?    5. How did you feel about having to be a part of the recent mass vaccination campaign? |
| Health Workforce | 1. Can you explain generally who are involved in providing immunization services?    1. In your opinion, were the current number of people sufficient during the mass vaccine campaign? 2. How has COVID affected the health workforce for providing immunization?    1. Are there any Health workers/FCHVs who dropped out from immunization roles due to COVID? Why? Why not? How were such challenges (staff’s security issues and unwillingness to work) managed?    2. How are the staff managed during COVID-19? (rotation, change of duties, etc.) |
| Health information System/Resources[[29]](https://liveunthsc-my.sharepoint.com/personal/sm1358_live_unthsc_edu/Documents/Smriti-Personal/Immunization%20Paper/IDI%20guidelines_7.22.2020.docx#_msocom_29) | 1. What resources are you using/following for educating patients or running awareness regarding COVID-19 nationwide?  2. What resources or guides do you use as a safety measure during COVID-19? (guide regarding social distancing, hand washing, masks etc.)  3. Can you describe the Health Information Management System to manage vaccine logistics during normal time and during COVID-19? |
| Financing | 1. Were there any additional funds allocated for providing services/managing health care workers/precautionary measures during COVID-19? Did you receive any additional incentives to take part in the mass vaccine campaign? |
| Leadership/governance | 1. Were you given any different training on how to safely provide vaccines this year as compared to last year?    1. What was different?    2. Was it helpful? Why? Why not? What could have made it better? 2. Were you provided with the personal protective equipment to safely provide the immunization services? 3. Are there any new governmental policies regarding service delivery during COVID-19? 4. Can you briefly tell me about how you feel regarding the preparedness measures taken by the higher authorities during this COVID?   a. Are there any mechanisms or system related to grievances to hear your concern regarding COVID-19? How do you feel about that?  b. Are there any policies in place regarding health service delivery during emergencies? Has there been any new changes in policies regarding health service delivery during COVID-19?  c. Could you briefly explain about the guidelines? For whom? What new ways are explained? |
| Recommendations/Way forward | 1. What should be the way forward in terms of preparing health care workers/FCHVs and the health systems to provide regular immunization services during a pandemic or any epidemic in future? 2. In your opinion, what could have helped you address the challenges faced due to COVID, especially in delivering regular immunization services? 3. Are there any other things about immunization services and pandemic that you would like to add? |

Thank you for your time and participation

**IDI for Parents**

| Name of the interviewee: |  |
| --- | --- |
| Age of the interviewee: |  |
| Occupation: |  |
| Date of Birth of Child or Age in Months |  |
| Educational Qualification of the Interviewee: |  |
| Name of the interviewer: |  |
| Date: |  |
| Time: |  |
| Start time: |  |
| End time: |  |
| How was the interview conducted? | Phone call / Zoom / Viber / Messenger /  WhatsApp / In-person |

|  | Guides |
| --- | --- |
| Icebreaker | 1. What is your opinion on the current COVID-19 pandemic? 2. How has it affected you? How has it changed your life? |
| Service Delivery | 1. In your opinion, how important is vaccination for your children? 2. Can you briefly describe the vaccines your child has received so far?    1. Normally, where do you take your child for vaccination?    2. When do you normally take your child for the vaccines?    3. Did your child ever miss the vaccines before the COVID-19? What happened? What did the health care providers do when the child missed the vaccination? 3. Has COVID-19 affected the health care service seeking behavior for your child? How? 4. When was the last time your child received the vaccines? Was it on a regular schedule? Can you explain the situation? 5. Did you take your child for the recent mass vaccination campaign?   *Yes, -* How did you find out about the mass vaccines? Can you explain how it was conducted?   - 1. What special precautions (taking temperature of everyone in the health facility/ social distancing/ use of PPEs) or provisions have been put in place to prevent staff and parents and children from COVID-19?   2. How did you feel about it? What could have been better?   *No-* Why not? What were your concerns? |
| Health Workforce | 1. Did your regular health care provider help you with any information in vaccination since covid? 2. Did you find services delivered by the health workforce satisfactory since covid and before covid? 3. Did your health care provider take any extra steps (wearing masks, using mobile phones) to help you with your child’s health condition? |
| Health information System/Resources | 1. How do you keep the records or track the vaccination schedule for your kids? 2. Did you receive any education about the vaccinations prior to COVID? Who gave you the information? 3. Did you receive any education about the vaccinations after the start of lockdown or COVID? Who gave you the information? What information did you receive? 4. If no, did you seek such information by yourself? Where did you find information and what sources were more reliable than others |
| Access to Vaccines | 1. Can you explain a normal vaccination visit to your health care provider?  a. Has there ever been a time before COVID when the vaccine was not available during the visit? If yes, can you tell us what happened?   b. What about after the lockdowns started?  c. How do you feel about it? |
| Leadership/governance | 1. In your opinion, has the government taken any steps to safely regulate immunization services during this pandemic? If yes, what are the steps? 2. If not, in your opinion, what could the government have done to safely regulate the immunization services for children during the pandemic? |
| Recommendations/Way forward | 1. What should be the way forward in terms of preparing parents to receive or health care providers to provide regular immunization services during a pandemic or any epidemic in future? 2. In your opinion, what could have helped you address the challenges faced due to COVID, especially in receiving regular immunization services for your child? 3. Are there any other things about immunization services and pandemics that you would like to add? |

Thank you for your time and participation
